# A novel high throughput screen to identify candidate molecular networks that regulate spermatogenic stem cell functions^†^

**DOI:** 10.1093/biolre/ioac048

**Published:** 2022-03-04

**Authors:** Tessa Lord, Nathan C Law, Melissa J Oatley, Deqiang Miao, Guihua Du, Jon M Oatley

**Affiliations:** School of Molecular Biosciences, Center for Reproductive Biology, College of Veterinary Medicine, Washington State University, Pullman, WA, USA; Priority Research Centre for Reproductive Science, Discipline of Biological Sciences, The University of Newcastle, Callaghan, NSW, Australia; Hunter Medical Research Institute, Infertility and Reproduction Program, New Lambton Heights, NSW, Australia; School of Molecular Biosciences, Center for Reproductive Biology, College of Veterinary Medicine, Washington State University, Pullman, WA, USA; School of Molecular Biosciences, Center for Reproductive Biology, College of Veterinary Medicine, Washington State University, Pullman, WA, USA; School of Molecular Biosciences, Center for Reproductive Biology, College of Veterinary Medicine, Washington State University, Pullman, WA, USA; School of Molecular Biosciences, Center for Reproductive Biology, College of Veterinary Medicine, Washington State University, Pullman, WA, USA; School of Molecular Biosciences, Center for Reproductive Biology, College of Veterinary Medicine, Washington State University, Pullman, WA, USA

**Keywords:** spermatogonial stem cell, regeneration, spermatogenesis, screen, high throughput, ID4, SRCAP, histone acetylation, ZSCAN2

## Abstract

Spermatogenic regeneration is key for male fertility and relies on activities of an undifferentiated spermatogonial population. Here, a high-throughput approach with primary cultures of mouse spermatogonia was devised to rapidly predict alterations in functional capacity. Combining the platform with a large-scale RNAi screen of transcription factors, we generated a repository of new information from which pathway analysis was able to predict candidate molecular networks regulating regenerative functions. Extending from this database, the SRCAP-CREBBP/EP300 (Snf2-related CREBBP activator protein-CREB binding protein/E1A binding protein P300) complex was found to mediate differential levels of histone acetylation between stem cell and progenitor spermatogonia to influence expression of key self-renewal genes including the previously undescribed testis-specific transcription factor ZSCAN2 (zinc finger and SCAN domain containing 2). Single cell RNA sequencing analysis revealed that ZSCAN2 deficiency alters key cellular processes in undifferentiated spermatogonia such as translation, chromatin modification, and ubiquitination. In *Zscan2* knockout mice, while spermatogenesis was moderately impacted during steady state, regeneration after cytotoxic insult was significantly impaired. Altogether, these findings have validated the utility of our high-throughput screening approach and have generated a transcription factor database that can be utilized for uncovering novel mechanisms governing spermatogonial functions.

## Teaser

A high-throughput RNAi screen was conducted to identify candidate molecules and molecular networks that control the regenerative function of spermatogonial stem cells.

## Introduction

Spermatogenesis is the process by which male gametes are produced that are ultimately destined to contribute genetic information to the next generation. Each sperm is genetically unique and in steady-state conditions, millions are generated daily from puberty until advanced age. The foundation of the spermatogenic lineage is the undifferentiated spermatogonial pool whose actions provide the basis for continuity and robustness of the system that occurs in asynchronized rounds (aka waves) throughout testicular parenchyma [1, 2]. A round of spermatogenesis initiates when a majority of the undifferentiated spermatogonial population (~95% in the mouse) transitions to a differentiating state in response to a pulse of retinoic acid, thus committing to a path of terminal differentiation as sperm [3, 4]. The remaining cells are tasked with regenerating the undifferentiated pool in preparation for a subsequent round of differentiating transition.

In addition to their key role in sustaining steady-state spermatogenesis, a minor portion of the undifferentiated spermatogonial population is responsible for regenerating the spermatogenic lineage following cytotoxic insults such as exposure to clastogens that deplete a majority of the spermatogenic lineage [5, 6]. Also, similar to other stem cell-based systems, a minor portion of the undifferentiated spermatogonial population is able to regenerate continual spermatogenesis following isolation from testicular tissue and then transplantation into seminiferous tubules of a recipient male or back into the donor male at a later date [6, 7]. Thus, regenerative capacity of a subset of the undifferentiated spermatogonial population (the spermatogonial stem cells, “SSCs”) is crucial not just for steady-state spermatogenesis but also for reestablishing the spermatogenic lineage in response to conditions of stress. Not surprisingly, impairment of this potential leads to permanent loss of the germline and infertility. At present, the core molecular mechanisms that influence the regenerative capacity of spermatogonia are largely undefined.

A bottleneck limiting in-depth exploration of the regenerative capacity of SSCs has been lack of knowledge about defining attributes and tools to study the population. In previous studies, we and others found that the regenerative potential in spermatogonia is linked to expression of the transcriptional repressor inhibitor of DNA binding 4 (ID4) [8–10]. Importantly, we developed an ID4-enhanced green fluorescent protein (*Id4-eGfp*) transgenic reporter mouse line that allows for assessing differences in the SSC content of spermatogonial populations following isolation from testicular tissue or after experimental manipulation in vitro [9, 10]. Within testes, spermatogonia with the highest levels of ID4 expression, distinguished as ID4-eGFP^Bright^ in the transgenic model, are a highly enriched, if not pure, population of stem cells [10]. In contrast, the population of spermatogonia with reduced expression of ID4, designated as ID4-eGFP^Dim^, possesses significantly less stem cell capacity and is in transition to becoming transit-amplifying progenitor spermatogonia that are marked as being ID4-eGFP-.

The transition from an SSC to transit-amplifying progenitor state in spermatogonia is characterized by a shift in gene expression, with RNAseq analyses revealing that over 1400 genes are differentially expressed between these subpopulations [9–11], yet the molecular mechanisms driving this transition are undefined. To address this gap in knowledge, we devised a high-throughput screening strategy with primary cultures of spermatogonia derived from *Id4-eGfp* transgenic mice and a large-scale transcription factor-targeting small interfering RNA (siRNA) library. Primary cultures of spermatogonia provide an invaluable resource for large-scale approaches by providing a tractable in vitro system in which SSCs and transit-amplifying progenitors largely retain their functional and gene expression signatures. Functional transplantation experiments have demonstrated that ID4-eGFP+ spermatogonia in primary cultures constitute a population with extensive regenerative capacity, whereas ID4-eGFP- cells represent the much more abundant progenitor pool that lacks stem cell capacity [9]. As a testament to the usefulness of this culture system, an abundance of novel genes that are now known to be integral for spermatogonial functions have been discovered in primary cultures before being further explored in vivo [9, 12–15]*.*

Here, we monitored fluctuation of the ID4-eGFP+ cellular content in primary spermatogonial cultures as a readout for our high-throughput screening platform, creating a database of information for over 1400 different transcription factors. From this information, previously unappreciated gene networks and associated pathways that are potential regulators of cell fate decisions and regenerative capacity in spermatogonia were identified including a role for histone acetylation that is mediated by the SRCAP-CREBBP/EP300 (Snf2 related CREBBP activator protein-CREB binding protein/E1A binding protein P300) complex. This complex was found to influence expression of the testis-specific transcription factor zinc finger and SCAN domain containing 2 (Zscan2). Furthermore, we found that ZSCAN2 plays an important role in maintenance of the SSC pool in vitro and is integral for robust regeneration of spermatogenesis in vivo following exposure to clastogens. Finally, using single cell RNA sequencing (scRNA-seq) we discovered that loss of ZSCAN2 expression alters key cellular processes in undifferentiated spermatogonia, such as translation, chromatin modification, and ubiquitin conjugation. Collectively, these findings demonstrate utility of the high-throughput screening approach and transcription factor database that was generated for making novel discoveries of mechanisms that influence spermatogenic stem cell functions.

## Materials and methods

### Animals

All animal procedures were approved by the Washington State Institutional Animal Care and Use Committee (IACUC). The *Id4-eGfp* mouse line was derived as described previously [9]. *Id4-eGfp* mice were crossed with *Rosa26LacZ* mice (Jackson Laboratories, stock number 112073), and spermatogonia from F1 generation were used to establish primary cultures. For spermatogonial transplantation analysis, F1 hybrids of C57BL6/J (Jackson Laboratories, stock number 000664) and 129S1/SvlmJ (Jackson Laboratories, stock number 112073) were used as recipients and pretreated with 55 mg/kg busulfan (Sigma, B2635) to ablate endogenous spermatogenesis, as described previously [16]. C57BL6/J mice were used as embryo donors for clustered regularly interspaced short palindromic repeats (CRISPR-Cas9) editing, and mutant knockout mouse lines were backcrossed on a C57BL/6 J background.

### Primary spermatogonial cultures

All cultures were established from Thy-1 cell surface antigen (THY1+) testis cell populations isolated from adult (3 months) or postnatal day (PD)6–8 testes and maintained in low oxygen conditions (10% O_2_) on mitotically inactivated SIM mouse embryo-derived thioguanine and ouabain resistant feeder monolayers (STOs) in mouse serum-fee medium (mSFM) supplemented with the growth factors glial cell–derived neurotrophic factor (GDNF) (20 ng/mL; R&D systems, Minneapolis, MN, USA) and fibroblast growth factor (FGF2) (1 ng/mL; BD Biosciences, San Jose, CA, USA), as described previously [10]. During maintenance of the cultures, i.e., before their utilization for the siRNA screen, media was changed every second day and cells were passaged every 6 days onto fresh feeders. For the siRNA screen, cells were isolated from established cultures (at passage 10 or higher), and experiments were completed before the culture reached passage 20 which is a point of in vitro aging that stem cell capacity is known to decline [17]. For experiments utilizing trichostatin A (TSA), cultures were incubated overnight with 10 μM TSA (Sigma, T8552) or dimethyl sulfoxide (DMSO).

### siRNA library transfection

**Figure 1:**
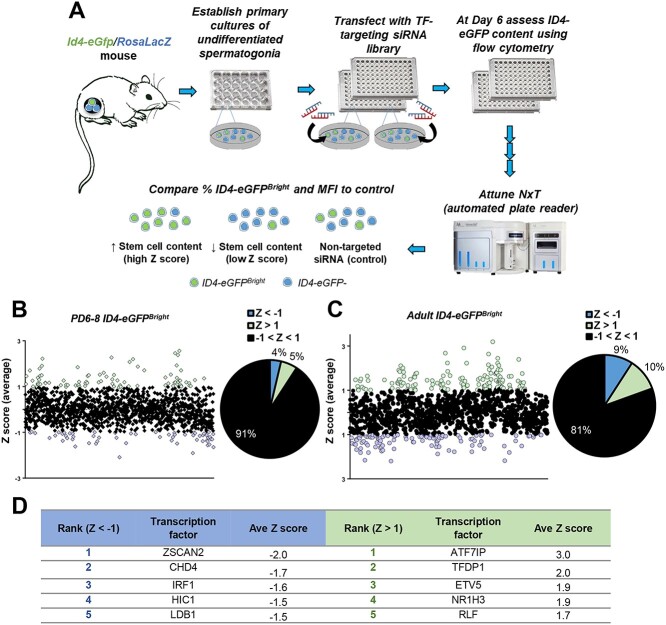
A large-scale siRNA screen to identify transcription factors that regulate spermatogonial activities. (*A*) Schematic of the experimental approach. Primary cultures of undifferentiated spermatogonia were established from *Id4-eGFP/Rosa LacZ* mice, and maintained in a 24-well plate. Spermatogonia were dislodged from the feeder layer, collected and transfected using siRNAs from a library of 1440 transcription factors (TFs) in a 96-well plate format. At 6 days post transfection, ID4-eGFP content was assessed using automated flow cytometry. For each transcription factor analyzed, an average Z-score was calculated that reflected fluctuations in the percentage of ID4-eGFP^Bright^ cells and MFI when compared with the nontargeted siRNA control. (*B*) Average Z-scores corresponding to ID4-eGFP^Bright^ content following knockdown of transcription factors in spermatogonial cultures established from PD6–8 mice (pups) (*n* = 3 independent cultures). Each data point corresponds to knockdown of one transcription factor. (*C*) Pie chart distribution of average Z-scores in pup cultures; 4% and 5% of factors had scores of Z < −1 and >1, respectively. (*D*) Average Z-scores corresponding to ID4-eGFP^Bright^ content following transcription factor knockdown in cultures established from adult mice (*n* = 3 independent cultures). (*E*) Pie chart distribution of average Z-scores in adult cultures; 9% and 10% of factors had scores of Z < −1 and >1, respectively. (*F*) List of transcription factors with the 5 lowest (blue) and 5 highest (green) Z-scores for ID4-eGFP^Bright^ content across all six replicates of the siRNA screen.

Transfection was carried out using protocols described in Cason and Lord [18]. Specifically, prior to transfection, cultured spermatogonia were isolated from feeder cells by gentle pipetting and distributed across a 96-well cell culture plate at a concentration of 20 000 cells/well. Transfection with siRNAs was facilitated using the Lipofectamine 3000 transfection reagent (ThermoFisher Scientific) at a volume of 0.4 μL/well. The siRNA library used for our large-scale approach was the “mouse siGENOME SMARTpool Transcription Factor library” (GE Dharmacon), which is comprised of 18 × 96 well plates, with 80 wells per plate containing a pool of four siRNAs targeted to a single transcription factor. Although it was not feasible to assess and optimize knockdown efficiency for each of the 1440 transcription factors in the siRNA library, we did utilize the fluorescent oligo “siGLO” (GE Dharmacon) to confirm that transfection efficiency was over 90% (Supplementary Figure S1C). Using this transfection strategy, we have previously demonstrated knockdown efficiencies of 50–80% for targets such as chromodomain helicase DNA binding protein 4 (*Chd4*) [19], RB transcriptional corepressor 1 (*Rb1*) [20], *Id4* [9], Neurogenin 3 (*Neurog3*) [21], and confirmed these trends via preliminary analyses using auxiliary siRNAs from our library (data for *Crebbp*, *Ep300*, and *Srcap* are provided in Supplementary Figure S1D). For transfection, 10 pmol of siRNA from the library was added to the corresponding culture well containing spermatogonia (“Day 0”). Additionally, four control wells were prepared at this time; a Lipofectamine 3000-only control, two wells transfected with a control nontargeted siRNA (GE Dharmacon), and one well transfected with siRNA targeted to *Rb1* to serve as an internal control. Transfection was conducted overnight (16 h), following which spermatogonia were treated with trypsin for 5 min, quenched with 10% fetal bovine serum (FBS), washed in mSFM, and plated back on feeder cell monolayers, maintaining their respective locations on a 96-well culture dish (“Day 1”). Transfected cultures were then maintained until Day 6, at which time they were prepared for flow cytometric analysis. A schematic of the transfection process with the siRNA library is provided in Figure 1A.

### Flow cytometric analysis

At 6 days after siRNA treatment, the ID4-eGFP content in primary cultures was assessed using the automated plate reader function of an Attune NxT flow cytometer (ThermoFisher). Forward and side scatter parameters, gating for the spermatogonial population, and identification of the eGFP+ subpopulations were conducted as described previously [9, 10, 19] and depicted in Supplementary Figure S1A. For assessment of H4K16ac (acetylation of lysine 16 on histone H4), cells collected from primary cultures via trypsin digest were fixed in 4% paraformaldehyde for 6 min at 37 °C, permeabilized in 90% methanol for 15 min on ice, washed once, and incubated overnight at 4 °C with an antibody recognizing H4K16ac (antibody information and dilution located in Supplementary Table S2). After two washes, secondary antibody incubations were carried out for 2 h at room temperature. Cell suspensions were then washed twice and analyzed by an Attune NxT flow cytometer.

### Spermatogonial transplantation

Spermatogonial transplantation was used to assess the regenerative capacity of each primary spermatogonial culture prior to initiating the siRNA screen (i.e., passage 10) and after the screen was completed (i.e., passage 18). For each culture and time point, cells were transplanted into six testes of three different recipient mice. In total, 72 recipient testes and 36 recipient mice were used to assess the stem cell capacity of six primary cultures used for the screen. Briefly, spermatogonial transplantation was conducted using methods described previously [6, 7, 22], with 10 000 cells being microinjected into each testis of a busulfan-treated recipient via the efferent duct. Two months after transplantation, recipients were euthanized and LacZ-expressing donor-derived colonies of spermatogenesis were visualized using X-Gal staining. Following colony counts, a “stem cell number” was determined by calculating the number of colonies produced per 10^5^ cells injected.

### Quantitative RT-PCR

RNA extraction was conducted using Trizol reagent per the manufacturer’s instructions (Invitrogen, CA, USA), followed by treatment with DNase I and reverse transcription using oligo d(T) priming and Superscript III reverse transcriptase (Invitrogen). Primer sequences for real-time quantitative reverse transcription PCR (qRT-PCR) are listed in Supplementary Table S1. Quantitative RT-PCR was conducted using an ABI 7500 fast sequence detection system (Applied Biosystems, Grand Island, NY, USA), and transcript abundance was normalized to the constitutively expressed ribosomal protein S2 (*Rps2*), calculated using the 2^−∆∆Ct^ formula, as described previously [23].

### Immunofluorescent staining

Immunofluorescent staining of testis cross-sections was conducted as described previously [24]. Briefly, paraffin-embedded testes were sectioned and mounted on slides. Slides were dewaxed with xylene and rehydrated in a series of ethanol washes (100, 90, 70, 30, and 0%) before 20 min antigen retrieval in boiling sodium citrate buffer (pH 6). Sections were blocked in serum for 1 h before overnight incubation in primary antibody (individual antibody dilutions provided in Supplementary Table S2). After three washing steps, secondary antibody incubations were carried out for 2 h at room temperature. Slides were mounted in Vectashield containing 4′,6-diamidino-2-phenylindole (DAPI) (Vector Laboratories, USA) and imaged on an IX 51 model inverted microscope (Olympus), and digital images were captured using a DP71 digital microscope camera and CellSense software (Olympus, Tokyo, Japan).

### Western blotting

Western blotting was conducted as described previously [24]. Briefly, protein lysates were collected using radioimmunoprecipitation assay (RIPA) buffer (Thermo Scientific) supplemented with protease inhibitor (Thermo Scientific) and phosphatase inhibitor (Sigma Aldrich) cocktails. Lysates were resolved on a 4–12% Bis Tris NuPAGE gel using an XCell SureLock Mini-Cell electrophoresis system (Invitrogen) and transferred onto nitrocellulose membrane. Membranes were blocked for 1 h with a 5% skim milk solution, diluted in Tris-buffered saline with 0.1% Tween (TBST). Primary antibody incubations were conducted overnight at 4 °C in 5% skim milk/TBST (antibody concentrations provided in Supplementary Table S2). Secondary antibody (goat anti-rabbit IgG HRP, Santa Cruz Biotechnology) was used at a concentration of 1/4000 for 1 h at room temperature. Blots were developed using enhanced chemiluminescence (ECL) prime detection reagent (GE Healthcare, PA, USA) and visualization on an LAS4000 imager (GE Healthcare).

### CRISPR-Cas9 generation of *Zscan2−/−* mice

Two guide RNAs (gRNAs) were designed for deleting a large portion of the protein coding region of the *Zscan2* gene (Supplementary Figure S6C and D). Single guide RNAs (sgRNAs) were selected based on off-target predictor scores provided by the online software programs CRISPOR (http://crispor.tefor.net/) and CRISPR Design (http://crispr.mit.edu/). sgRNAs with an off-target predictor score of >80 were then further screened based on the following criteria: (1) proximity to the start codon, (2) adjacent to protospacer adjacent motif (PAM) sequence in sense and antisense directions, and (3) homologous targets in both alleles. Selected sgRNAs targeting *Zscan2* were then generated (sequences provided in Supplementary Figure S6D). PCR (using DreamTaq, Thermo Fisher Scientific, Waltham, MA, USA) was firstly used to create two overlapping oligonucleotides: (1) CRISPR (GAAATTAATACGACTCACTATAGGN_18-20_*GTTTTAGAGCTAGAAATAGC*) that incorporates sequence specific for the desired target site, the GGN20 guide sequence, and T7 promoter sequence; and (2) a common oligonucleotide that contains the sgRNA stem loop structure for docking of Cas9. The resultant single-stranded DNA (ssDNA) was purified using a Qiaquick Gel Purification kit (Qiagen, Hilden, Germany) and used as a template for synthesis of sgRNA with the MEGAshortscriptTM Kit (Thermo Fisher Scientific, Waltham, MA, USA). Mature sgRNAs were cleaned up (Turbo Dnase, Thermo Fisher Scientific, Waltham, MA, USA) and purified using the MEGAclear™ Kit (Thermo Fisher Scientific, Waltham, MA, USA) and eluted at ~2000 ng/μL in TE buffer (10 mM Tris–HCL, 0.1 mM EDTA, pH 7.4). Resultant sgRNAs were stored in aliquots at −80 °C for up to 6 months.

For generation of mice, a microinjection solution was created that contained sgRNAs (100 ng/μL each) and Cas9 mRNA (200 ng/μL) (TriLink Biotechnologies, San Diego, CA, USA) diluted in Tris-EDTA (TE) buffer at 1:1 mass ratio and then incubated at room temperature for 10 min. Opti-MEM Reduced Serum media with no phenol red (Gibco/Thermo Fisher Scientific, Waltham, MA, USA) was then added to provide a final reaction volume of 20 μL. Microinjected zygotes were surgically transferred into pseudo-pregnant recipient females. A founder male carrying a 245 bp deletion (frameshift mutation) allele was produced (Supplementary Figure S6C–F) and used to establish a *Zscan2* edited mouse line.

### Single cell RNA-sequencing

To generate enriched spermatogonial populations for scRNA-seq, testes from three PD8 wild type mice, and three PD8 *Zscan2−/−* mice were digested into single cell suspensions and subjected to THY1+ magnetic-activated cell sorting (MACS) selection according to the manufacturer’s protocol, and as described previously [17]. Equal numbers of THY1+ spermatogonia from each wild-type mouse were combined into a single population for complementary DNA (cDNA) library preparation, and an equivalent population was created using pooled *Zscan2−/−* spermatogonia. These populations of undifferentiated spermatogonia were also “spiked” with unselected cells from the MACS preparation, to ensure that the differentiating spermatogonia pool was also captured in our single-cell analysis. Live cells from wild type and *Zscan2−/−* mice were loaded into a Chromium Controller (10X Genomics, Inc.), and single-cell cDNA libraries were generated as per the manufacturer’s instructions. Wild-type and *Zscan2−/−* libraries were pooled and sequenced in a single lane on an Illumina HiSeq 4000 (Genomics and Cell Characterization Core Facility, University of Oregon). Raw base call files were demultiplexed using the 10X Genomics Cell Ranger pipeline and aligned to the mouse mm10 transcriptome.

Wild-type and *Zscan2−/−* transcriptomes were imported into Seurat and merged into a single object [25]. Doublets and cells with low-quality transcriptomes were filtered from the dataset, and testicular somatic cells were excluded based on expression of previously characterized markers for each subpopulation [26]. The data was then normalized and scaled using Seurat. The “FindVariableGenes” function was used to identify variable genes for use in principle component analysis. For clustering and t-distributed stochastic neighbor embedding (*t*-SNE) graphing, 14 significant principal components were used (resolution set to 0.5).

### Data analyses

For the siRNA screen, six different primary cultures of spermatogonia were utilized as biological replicates; three derived from PD6–8 and three from adult (>2 months of age) mice. In each replicate, normalized values were produced using readout from the nontargeted siRNA control. A Z-score was then calculated using the formula: [*Z* = (*X* − *μ*)/*σ*], where *X* is the normalized value for each data point, *μ* is the mean, and *σ* is the standard deviation (calculated using GraphPad Prism software, version 6 (La Jolla, CA, USA)). For qualitative analyses of siRNA screen output, the DAVID Bioinformatics Resources (V6.8) [27, 28] and STRING (V10.5) [29] platforms were utilized. For these analyses, lists of candidates scoring either Z > 1 or Z < −1 were used.

All additional experiments were conducted with a minimum biological replication of three. All quantitative data is presented as Mean ± standard error of the mean (SEM). Differences between means were determined statistically using the one-way analysis of variance (ANOVA) or *t*-test function of GraphPad Prism 6 software. Multiple comparison analysis was carried out using the Tukeys post-hoc test function of GraphPad. A value of *P* < 0.05 was considered to be statistically significant.

## Results

### Design of a high-throughput screen to assess fluctuations in regenerative capacity

To predict alterations in the regenerative capacity of the undifferentiated spermatogonial population on a large scale, we devised a high-throughput screening approach with primary cultures of spermatogonia established from either prepubertal pup or adult *Id4-eGfp* transgenic mice (Figure 1A). The approach utilizes flow cytometric analysis (FCA) to measure alterations in the ID4-eGFP+ content of the cultured cell population. To quantitate changes in the proportion of SSCs (ID4-eGFP^Bright^), cells transitioning to the progenitor state (ID4-eGFP^Mid/Dim^), and cells in the transit-amplifying progenitor state (ID4-eGFP-), the eGFP profile of the cultured spermatogonial population was divided into quadrants on a scatter plot (Supplementary Figure S1A). To validate the readout, we monitored changes in the percentage of ID4-eGFP^Bright^ cells and mean fluorescent intensity (MFI, a reflection of overall changes in the ratio of stem cell, transitory, and progenitor spermatogonia) of the entire cultured population in response to siRNA knockdown of retinoblastoma protein (*Rb1*) expression (Supplementary Figure S1B), a molecule that is required for maintenance of the regenerative spermatogonial population [20, 30]. Treatment conditions were optimized to achieve >95% siRNA transfection efficiency (Supplementary Figure S1C) and a 50–80% knockdown efficiency (Supplementary Figure S1D), and considering that the self-renewal rate of stem cells in primary cultures of spermatogonia is ~5.6 days [31], we assayed the ID4-eGFP profile 6 days after transfection. As expected, the proportion of the cell population that was measured as ID4-eGFP^Bright^ was significantly (*P* < 0.01) reduced by 32 ± 2.0% (mean ± SEM, *n* = 4 different cultures) in *Rb1* siRNA treated cultures compared with control cultures treated with a nontargeting siRNA; whereas the proportion of the population that was ID4-eGFP^Mid^ or ID4-eGFP^Dim^ was not different between *Rb1* siRNA and control cultures (Supplementary Figure S1B). Similarly, the MFI of the culture overall was significantly reduced (*P* < 0.001) by 40 ± 3.2% (mean ± SEM, *n* = 8 different cultures) (Supplementary Figure S1B). Both levels of reduction (ID4-eGFP^Bright^ content and MFI) align with the decrease in regenerative capacity of *Rb1* siRNA treated cultures measured previously by functional transplantation [20]. These data validate the approach of using alteration in ID4-eGFP content of primary spermatogonial cultures as a readout of altered SSC maintenance. In the course of carrying out these validation experiments, we found that taking into account both alteration in the ID4-eGFP^Bright^ population and MFI provided an optimal readout from siRNA mediated knockdown of gene expression (Supplementary Figure S1B). Therefore, further analyses focused on a combination of these measures to identify transcription factor networks influencing regenerative capacity of cultures. However, raw data depicting fluctuations in the percentage of total ID4-eGFP+, eGFP^Dim^, and eGFP^Mid^ cells, as well as changes in cell viability have been provided in Supplementary Dataset S1.

Using the optimized methodology for siRNA transfection and FCA of the ID4-eGFP profile in cultures, we next aimed to conduct a large-scale high throughput screen of transcription factors to identify potentially novel regulators of spermatogenic stem cell capacity. To achieve this, we used the mouse siGENOME SMARTpool Transcription Factor Library from Dharmacon Inc. that includes pooled siRNAs for 1440 transcription factors (Figure 1A). To provide comprehensive coverage, the screen was conducted with six different primary cultures of spermatogonia; three cultures derived from PD6–8 mice (designated as “pup cultures”), and three cultures derived from adult (3 months of age) mice (designated as “adult cultures”). Importantly, stem cell content in all cultures was confirmed via spermatogonial transplantation at the beginning and end of the screening process (Supplementary Figure S1E and F). At the conclusion of the siRNA screen, an average Z score was calculated for each data point (i.e., transcription factor) pertaining to each experimental parameter; i.e., ID4-eGFP^Bright^ content and MFI (Figure 1B–E and Supplementary Figure S2A and B). As expected, knockdown of a majority of transcription factors analyzed did not significantly disrupt the dynamics of the cultured population; however, at a threshold of Z > 1 and Z < −1, a significant number of candidate regulators were identified based on the knockdown causing a reduction or increase in ID4-eGFP^Bright^ content or MFI. In evaluating the ID4-eGFP^Bright^ dataset from pup cultures, 5% of transcription factors fell in the Z > 1 range and 4% in the Z < −1 range (Figure 1C). In adult cultures 10 and 9% of factors were in the Z > 1 and Z < −1 ranges, respectively (Figure 1E). For MFI analyses, 5 and 9% of transcription factors were in the Z > 1 range for pup and adult cultures, respectively, whereas 2 and 4% of transcription factors were in the Z < −1 range for pup and adult cultures, respectively (Supplementary Figure S2A and B). Collectively, this large-scale database provides a key resource for identifying potential novel regulators and transcription factor networks driving regenerative capacity in spermatogonia. The entire raw dataset is available for download as Supplementary Dataset S1.

From the database of information generated by the screen, we aimed to identify a list of prime candidate transcription factors that potentially regulate fate decisions in spermatogonia. To achieve this, the top 5 factors with the highest and lowest average Z scores across all six biological replicates of the screen based on alterations in ID4-eGFP^Bright^ content (Figure 1F) or MFI (Supplementary Figure S2C) were selected. In total, 18 factors were identified from this combined filtering that included ETS variant 5 (*Etv5)* and *Rb1* both of which have been demonstrated previously to be important for stem cell function in spermatogonia [20, 32], and *Chd4*, which we have recently demonstrated to be integral for SSC maintenance [19]. The remaining 15 factors have not been described in previous studies as regulators of spermatogonial functions and therefore represent novel candidates that potentially influence maintenance of stem cell capacity, transition to a progenitor state, or regenerative capacity.

### Identification of putative transcription factor networks that influence stem cell capacity in spermatogonia

**Figure 2:**
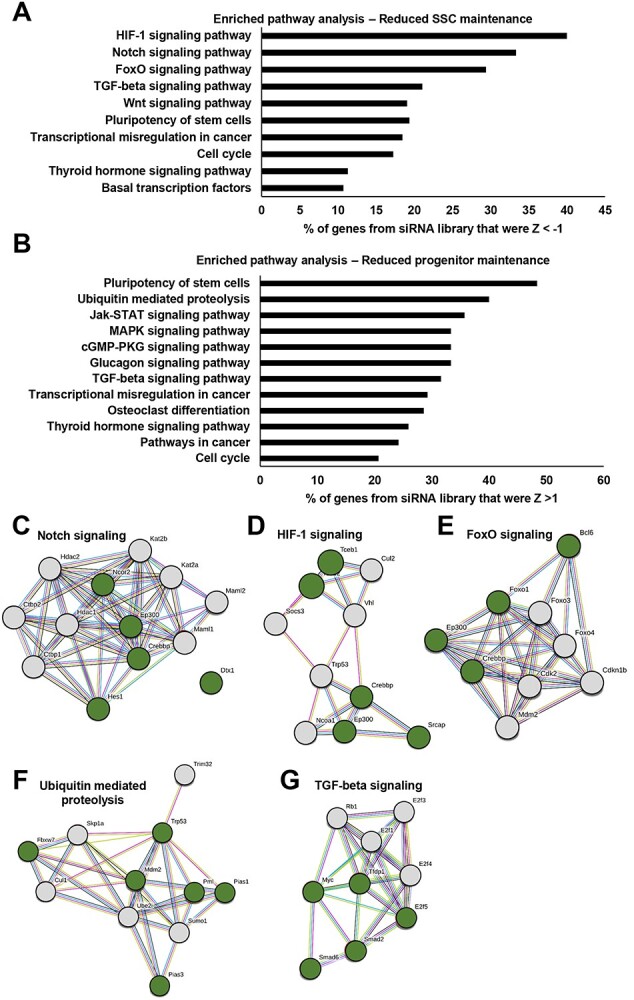
Pathway analysis of transcription factors flagged as being important for maintenance of stem cell content in primary cultures of undifferentiated spermatogonia. (*A* and *B*) Significantly enriched pathways identified from DAVID bioinformatics analysis of transcription factors scoring Z < −1 (A) or Z >1 (*B*) in ID4-eGFP^Bright^ content and MFI. Values are the percentage of transcription factors in each category that scored Z < −1 or Z > 1 in comparison to all transcription factors of the full siRNA library. (*C*–*E*) STRING analysis to assess interaction among top-ranking pathways for Z < −1 factors; Notch signaling (*C*), HIF-1 signaling (*D*), and FoxO signaling (*E*). Green circles denote proteins in the network that scored Z < −1 in the siRNA screen. Common to these pathways was a core cluster of transcription factors that comprise or interact with the CREB acetylation complex, i.e., CREBBP, EP300, and SRCAP. (*F* and *G*) STRING analysis to assess interaction among top-ranking pathways for Z > 1 factors; ubiquitin-mediated proteolysis (*F*), and TGF-beta signaling (*G*). Green circles denote proteins in the network that scored Z > 1 in the siRNA screen.

To gain deeper insight, we sought to use the siRNA screen database for identifying interactive networks that potentially influence the maintenance of stem cell capacity in spermatogonia. To achieve this, the dataset was filtered for all genes with a Z score of >1 and < −1 based on alterations in the ID4-eGFP^Bright^ population and MFI of the culture overall which yielded 547 factors. From this list, we firstly used the DAVID Bioinformatics Resources platform to identify pathways that potentially regulate stem cell maintenance (i.e., we analyzed factors whose knockdown in expression led to a reduction in the ID4-eGFP^Bright^ population and MFI (Z < −1)). The top 10 significantly enriched pathways are listed in Figure 2A. From these, we chose Notch, hypoxia-inducible factor-1 (HIF-1), and forkhead box transcription factor (FOXO) signaling for further analysis using the STRING platform (Figure 2C–E, Supplementary Dataset S1). Interestingly, a subset of candidate factors were found to be present in all three protein networks, namely CREBBP (a “top 5” prime candidate flagged in Supplementary Figure S2C) and EP300, which are core members of the CREB acetylation complex. In addition to these molecules, SRCAP and forkhead box O1 (FOXO1) which are also known to participate in the CREB-mediated acetylation complex were present in the protein network atlases for HIF-1 and FOXO signaling. To validate these bioinformatic-based findings, we further analyzed the impact of knocking down expression of CREBBP, EP300, SRCAP, and FOXO1 in primary cultures of undifferentiated spermatogonia on changes in the dynamics of the ID4-eGFP subpopulations (Supplementary Figure S3A and B). Outcomes revealed a significant (*P* < 0.001) decline of the ID4-eGFP^Bright^ population compared with cultures treated with nontargeting control siRNA for all factors (Supplementary Figure S3A and B), in the absence of any significant changes in cell viability (Supplementary Dataset S1). Taken together, these findings support a model in which the CREB complex and interacting proteins are involved in acetylation of genes that influence maintenance of the stem cell state in spermatogonia.

As a complement to experiments described above, we also investigated the list of candidate transcription factors with a Z score of >1 that potentially drive the stem cell-to-progenitor transition (i.e., factors whose knockdown in expression led to an increase in the ID4-eGFP^Bright^ population and MFI) using the DAVID platform. The top 10 significantly enriched pathways are listed in Figure 2B. Notably, this analysis yielded pathways regulating ubiquitin-mediated proteolysis, transforming growth factor (TGF)-beta signaling, cell cycle progression, and pluripotency. In addition, STRING analysis identified interacting protein complexes, particularly in the categories of ubiquitin mediated proteolysis (Figure 2F, Supplementary Dataset S1) and TGF-beta signaling (Figure 2G, Supplementary Dataset S1).

Of particular interest was the top-ranking candidate in the ID4-eGFP^Bright^ dataset (Figure 1F), activating transcription factor 7 interacting protein (ATF7IP), a transcriptional co-repressor/activator with genetic polymorphisms that have been linked to germ cell tumor formation in humans [33]. Interestingly, the percentage of ID4-eGFP^Bright^ cells in both adult and pup cultures was significantly (*P* < 0.001) increased by >2-fold in cultures subjected to *Atf7ip* knockdown compared with the nontargeted siRNA control cultures (Supplementary Figure S3C and D). The total number of ID4-eGFP^Bright^ cells was 2-fold greater in *Atf7ip* knockdown cultures; whereas the number of cells in the other subpopulations (i.e., eGFP-, eGFP^Dim^, and eGFP^Mid^) was not significantly altered (Supplementary Figure S3C and D), suggesting that proliferation within the stem cell pool may have been impacted. Interestingly, methyl-CpG binding domain protein 1 (MBD1), a known binding partner of ATF7IP, was also flagged in the Z > 1 screen database. Further assessment of the dataset revealed that *Mbd1* knockdown also led to a significant (*P* < 0.001) increase by 2-fold in the percentage of ID4-eGFP^Bright^ cells in primary cultures derived from adult mice; however, this trend was not observed in pup cultures (Supplementary Figure S3C). Taken together, these data suggest that ATF7IP working in concert with MBD1 influences the stem cell-to-progenitor transition and/or stem cell proliferation in spermatogonia and demonstrate that future investigations using our database as a resource should consider the effect of transcription factor knockdown on proliferation/mitotic index.

### Comparison of responses in pup versus adult spermatogonia to transcription factor knockdown

Spermatogonial populations in testes of prepubertal pups and adult mice have seemingly different properties, owing to their trajectories of establishing the spermatogonia pool and driving the first wave of spermatogenesis, versus maintaining steady-state spermatogenesis, respectively [10, 26]. Thus, we next sought to use the database generated by the large-scale screen to identify commonality and uniqueness in transcription factors that potentially influence functional capacities. To achieve this, changes in Z score (∆Z) for all 1440 transcription factors analyzed based on alterations in the ID4-eGFP^Bright^ content (Supplementary Figure S4A) and MFI (Supplementary Figure S4B) were used to compare adult and pup cultures. The mean ∆Z for both parameters was ~0.59 and knockdown of ~84% of transcription factors caused a similar response in pup and adult cultures (i.e., ∆Z score was <1); whereas knockdown of ~16% of transcription factors produced a ∆Z score of >1. Filtering for a ∆Z score of >2 yielded 21 and 25 transcription factors whose knockdown produced a significantly different response in pup versus adult cultures for the parameters of ID4-eGFP^Bright^ content (Supplementary Figure S4C) and MFI (Supplementary Figure S4D), respectively. To explore these further, we combined the lists and conducted pathway analysis (Supplementary Figure S4E, Supplementary Dataset S1). Enriched pathways included those regulating pluripotency of stem cells and transcriptional misregulation in cancer. The differential response of pup versus adult spermatogonia to the knockdown of a small number of transcription factors aligns with findings from previous scRNAseq analyses, in which a suite of differentially expressed genes (DEGs) was identified between SSCs at these age points [26]. Combined, our data and previously published RNAseq datasets [26] will provide an interesting starting point for future investigations into age-related differences in spermatogonia function.

### Use of the screen database to identify the CREB complex as a mediator of differential histone acetylation in stem cell and progenitor spermatogonia

**Figure 3:**
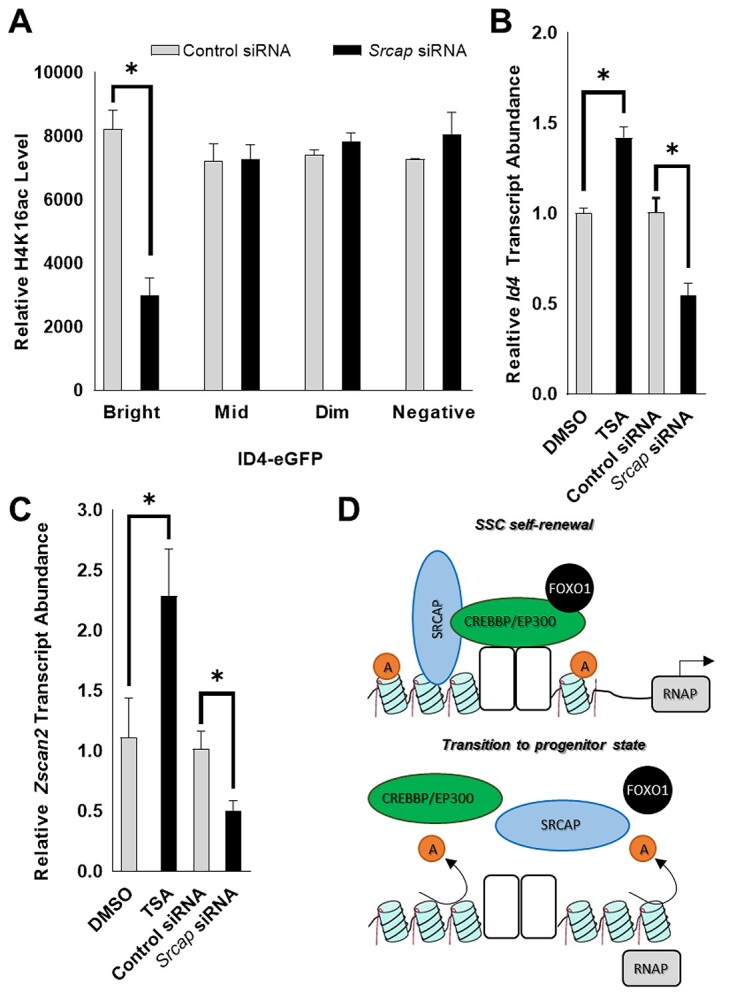
Influence of H4K16ac levels on the stem cell state in spermatogonia. (*A*) Relative levels of H4K16ac in ID4-eGFP expressing subpopulations of primary cultures of undifferentiated spermatogonia treated with nontargeting control or *Srcap* siRNA. The *y*-axis values are mean fluorescent intensity units derived from flow cytometric analysis with an antibody that recognizes H4K16ac. (*B* and *C*) Relative abundance of *Id4* (*B*) and *Zscan2* (*C*) transcripts in primary cultures of undifferentiated spermatogonia following treatment with TSA or *Srcap* siRNA to increase or decrease H4K16ac levels, respectively. Nontargeting siRNA or DMSO was used for controls. Data are derived from qRT-PCR analyses. Data in *A*–*C* are mean ± SEM for *n* = 3 different cultures and ^*^ denotes significantly different at *P* < 0.05. (*D*) Proposed role of the CREB acetylation complex and interacting proteins on influencing functional capacities in spermatogonia.

In light of the screen outcomes flagging the CREB complex components SRCAP, CREBBP, and EP300, which are all highly expressed in SSCs based on previously published RNAseq data [10], we next aimed to explore whether their known role of modulating histone H4 lysine 16 acetylation (H4K16ac) in other cell types [34] is also at play in spermatogonia. First, using FCA with primary cultures of spermatogonia established from *Id4-eGfp* transgenic pups, we found that the overall level of H4K16ac was greater by ~12.1 ± 0.9% (mean ± SEM, *n* = 3 different cultures) in the SSC-enriched ID4-eGFP^Bright^ population compared with the ID4-eGFP^Mid^ and ID4-eGFP^Dim^ counterpart populations (Figure 3A). In addition, treatment of the cultures with TSA which increases overall histone acetylation through inhibition of histone deacetylases led to an increase in the proportion of the population that was ID4-eGFP^Bright^ and ID4-eGFP^Mid^ with a concomitant decline in the ID4-eGFP- population (Supplementary Figure S5B), thus suggesting that histone acetylation levels overall influence the progenitor-to-SSC transition. Second, focusing on SRCAP as the prime activator of the complex, we found that knockdown of expression by siRNA treatment led to a significant (*P* < 0.05) reduction of >2.7-fold in H4K16ac levels of the ID4-eGFP^Bright^ population specifically without altering levels in either the ID4-eGFP^Mid^ or ID4-eGFP^Dim^ populations (Figure 3A). In addition, TSA treatment caused a significant (*P* < 0.05) increase in *Id4* gene expression whereas *Srcap* siRNA treatment resulted in significant (*P* < 0.05) reduction (Figure 3B). Moreover, a similar response was measured for expression of *Zscan2* (Figure 3C), which was the top-ranking Z < −1 prime candidate identified from the screen (Figure 1F). Collectively, these findings suggest that CREB complex (in particular SRCAP) mediated H4K16ac levels in spermatogonia may influence stem cell and progenitor states (Figure 3D). However, additional analyses, such as spermatogonial transplantation following knockdown of CREB complex components, should be the focus of future studies to consolidate these findings.

### Selection of *Zscan2* as a prime candidate for in vivo functional exploration

**Figure 4:**
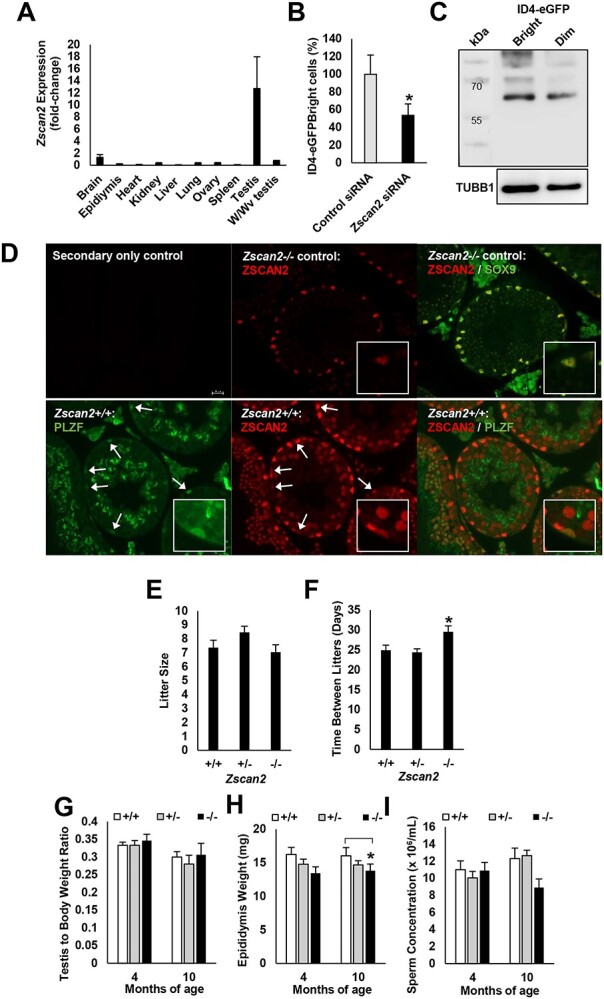
Impact of ZSCAN2 deficiency on steady-state spermatogenesis. (*A*) Quantitative PCR analysis of *Zscan2* transcript abundance in different tissues of adult mice. The expression level in testes was >10-fold higher compared with all other tissues analyzed. Data are mean ± SEM for *n* = 3 different mice. (*B*) ID4-eGFP^Bright^ content in primary cultures of undifferentiated spermatogonia treated with *Zscan2* or nontargeted control siRNA. Data are mean ± SEM for *n* = 6 different primary cultures and ^*^ denotes significantly different at *P* < 0.05. (*C*) Western blot analysis of relative ZSCAN2 protein (~67 kDa, arrow) levels in ID4-eGFP^Bright^ and ID4-eGFP^Dim^ subpopulations in PD8 mouse testes. Tubulin was used as a loading control. (*D*) Immunofluorescent staining for ZSCAN2 (red) in cross-sections of adult mouse testes. Intense staining is evident in undifferentiated spermatogonia (PLZF+, green cells highlighted by white arrows, lower images) and in spermatocytes and round spermatids. Staining in Sertoli cells (SOX9+, green, upper images) was found to be nonspecific, as fluorescence persisted in *Zscan2−/−* testis sections. Magnified images provided in inset. (*E*–*F*) Fertility assessment of *Zscan2−/−* male mice in comparison to wild type (*Zscan2+/+*), and heterozygous (*Zscan2+/−)* littermates. Data are mean ± SEM for *n* = 3 different male mice each genotype paired with two wild-type females every 2 months over a 10-month period and ^*^ denotes significantly different at *P* < 0.01. (*G*–*I*) Comparison of testis weight, epididymal weight, and sperm production in *Zscan2+/+*, *Zscan2+/−*, and *Zscan2−/−* male mice at 4 and 10 months of age. Data are mean ± SEM for *n* = 3 mice of each genotype at each age and ^*^ denotes significantly different at *P* < 0.05.

Next, we sought to explore the utility of information generated by the screen for identifying novel regulators of regenerative capacity in spermatogonia by conducting in-depth analysis of a single factor. To select a prime candidate, a multistage process was employed. First, we filtered out the top 5 transcription factors with a Z score of <−1 for both changes in ID4-eGFP^Bright^ content and MFI. Second, we searched GEO databases for the expression profile of each factor to identify any that are expressed highly or exclusively in testes. Third, factors that had not been previously characterized as being expressed in spermatogonia were given priority. This process yielded *Zscan2* as a prime candidate, and considering that its expression level in primary cultures of spermatogonia was found to be influenced by SRCAP-mediated H4K16ac levels, we selected it for in-depth analysis. The amino acid sequence of ZSCAN2 is highly conserved among mammalian species (Supplementary Figure S6A), and based on transcriptome profiling, gene expression is restricted to the brain and testis [35]. Using qRT-PCR analysis, we found that *Zscan2* gene expression in the testis is >10-fold higher compared with the brain and undetectable in other tissues of mice (Figure 4A). In addition, *Zscan2* transcript abundance was found to be greatly reduced in testes of W/Wv mice that are depleted of endogenous germline but contain somatic cells [36, 37], indicating that expression in the testis is largely attributed to germ cells (Figure 4A). In primary spermatogonial cultures derived from either pup or adult mice, knockdown of *Zscan2* by siRNA treatment resulted in a reduction of ID4-eGFP^Bright^ cell content by ~50% compared with control cultures treated with nontargeting siRNA (Figure 4B). Moreover, based on Western blot analysis of FACS isolated cell populations from testes of PD8 mice, ZSCAN2 protein was detectable in both ID4-eGFP^Bright^ and ID4-eGFP^Dim^ spermatogonia (Figure 4C). Localization of ZSCAN2 in cross-sections of adult mouse testes by immunofluorescent staining further confirmed expression by spermatogonia as well as spermatocytes and round spermatids (Figures 4D, Supplementary Figure S6B). ZSCAN2 expression (red fluorescence) within undifferentiated spermatogonia was again demonstrated, this time via co-staining with an anti-promyelocytic leukemia zinc finger (PLZF) antibody (green fluorescence) (Figure 4D, lower images), while antibody staining in Sertoli cells (SOX9+, green, Figure 4D, upper images) was shown to be nonspecific based on continued antibody reactivity in *Zscan2−/−* sections. Expression of ZSCAN2 by spermatogonia was also detectable in cross-sections of prepubertal (3–4 months of age) bovine and porcine testes (Supplementary Figure S6B), and can be observed in cross-sections of adult human testes [Human Protein Atlas, https://www.proteinatlas.org/ENSG00000176371-ZSCAN2/tissue/testis#img, [38]]. Further, >88% homology in the amino acid sequences for ZSCAN2 exists between mouse, human, bovine, and porcine (Supplementary Figure S6A). Considering the highly conserved nature of *Zscan2* as a testis-enriched gene in mammalian species, we chose to further explore the role of this molecule in the regulation of spermatogenesis.

### Generation of a *Zscan2* knockout mouse line

A mutant animal model of impaired *Zscan2* function had not been reported in the literature, thus we generated a knockout mouse line using CRISPR-Cas9 technology. To achieve this, a dual guide RNA (gRNA) approach was devised to target exon 2 of the murine *Zscan2* gene (Supplementary Figure S6C and D). These gRNAs were combined with Cas9 mRNA and introduced into zygote stage embryos from C57BL6/J mice followed by transfer to pseudo-pregnant recipients*.* From these transfers, a founder male containing a 245 bp deletion allele in a region of the gene that is common to all known transcript variants, and that disrupted the reading frame of *Zscan2*, was selected for building a line on a C57BL6/J genetic background in which homozygous animals lacked expression of ZSCAN2 (Supplementary Figure S6E and F, Figure 4D). The *Zscan2* deletion allele was transmitted at an expected Mendelian inheritance. Both *Zscan2+/−* and *Zscan2−/−* animals were healthy and thrived into adulthood. Outcomes of a 10 month breeding trial revealed no difference in fecundity between *Zscan2+/+*, *+/−*, and *−/−* littermates (Figure 4E), although the time between litters was significantly longer (*P* < 0.01) for wild-type females that were paired with *Zscan2−/−* males when compared with those paired with *Zscan2+/−* or *+/+* males (Figure 4F). Accordingly, at 4 and 10 months of age the testis to body weight ratio was not found to be different between *Zscan2−/−*, *+/−*, and *+/+* males (Figure 4G); however, the paired epididymal weight was significantly (*P* < 0.05) reduced for *Zscan2−/−* males compared with *Zscan2+/+* controls (Figure 4H). Additionally, relative sperm concentration in the caudal epididymis was reduced by ~18% in *Zscan2−/−* males compared with *Zscan2+/+* control males at 10 months of age (*P* = 0.09, assessed via mincing of the caudal epididymides and calculating the number of sperm per mL of diluent) (Figure 4I). Taken together, these findings suggest that while mice heterozygous for the *Zscan2* deletion exhibit a reproductive phenotype identical to that of wild-type controls, homozygous *Zscan2* deletion causes a minor quantitative disruption of spermatogenesis during homeostatic conditions.

### ZSCAN2 is required for normal regeneration of the spermatogenic lineage following cytotoxic insult

**Figure 5:**
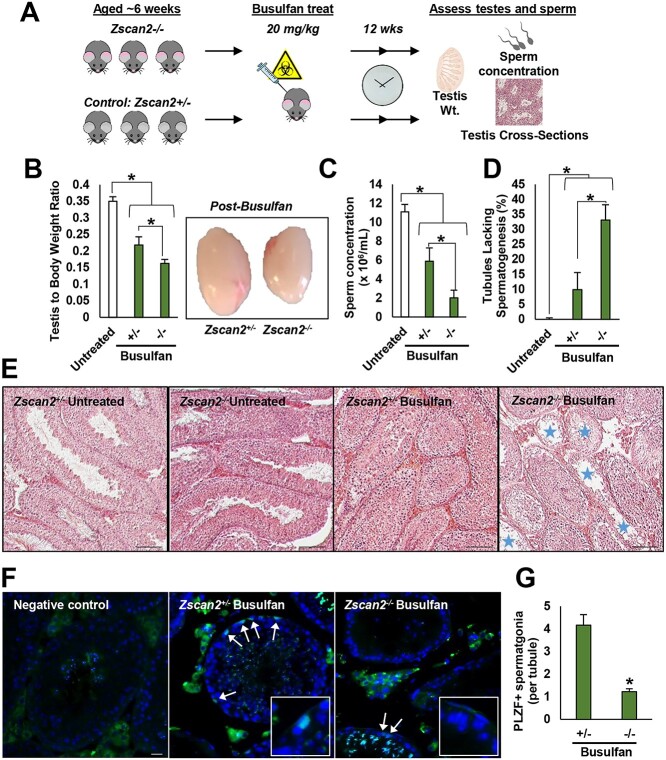
Impact of ZSCAN2 deficiency on regenerative capacity of the spermatogenic lineage following clastogen exposure. (*A*) Schematic of the experimental strategy. (*B*–*G*) Spermatogenic phenotype at 12 weeks post busulfan treatment for Zscan2−/− and Zscan2+/− mice. (*B* and *C*) Testis to body weight ratio and caudal epididymal sperm concentration (*C*); “untreated” controls shown in bar graphs are Zscan2+/− mice, which are not statistically different from Zscan2−/− and Zscan2+/+ at this age point (refer to Figure 5G–I), data are mean ± SEM. for *n* = 4 mice of each genotype and ^*^ denotes significantly different at *P* < 0.05. (*D* and *E*) Representative images of testicular cross-sections stained with Hematoxylin and Eosin and quantification of the percentage of seminiferous tubules lacking spermatogenesis. Blue stars denote seminiferous tubule cross-sections that are devoid of spermatogenesis. Data in D are mean ± SEM for *n* = 4 mice of each genotype and five different cross-sections per testis and ^*^ denotes significantly different at *P* < 0.05. (*F* and *G*) The number of undifferentiated spermatogonia (PLZF+, green fluorescent cells, highlighted by white arrows) per tubule was calculated via immunofluorescence analysis. Data are mean ± SEM. for *n* = 4 mice of each genotype and ^*^ denotes significantly different at *P* < 0.001.

In nonhomeostatic conditions, the sustainment of fertility following exposure to environmental stressors such as clastogens relies on efficient regeneration of the spermatogenic lineage that initiates with the activities of the undifferentiated spermatogonial population. It can be postulated that cultured SSCs may align with SSCs in regenerative conditions in vivo, given that they are passaged every 6 days and are thus tasked with proliferation to repopulate the culture dish. We speculated that this may explain the discrepancy between our findings following *Zscan2* knockdown in culture when compared with homeostatic conditions in the *Zscan2* knockout mouse. Thus, to assess whether ZSCAN2 is functionally important for SSC function in regenerative conditions in vivo, we designed an experimental scheme that included exposing *Zscan2−/−* and *+/−* males to a low dose (20-mg/kg body weight) of the chemotoxic drug busulfan and assessing spermatogenesis several months later (Figure 5A). In wild-type males, exposure to busulfan causes depletion of the endogenous germline and at low doses the effect is transient with complete regeneration after a timeframe that would constitute one or two rounds of spermatogenesis. Although spermatogenesis was mildly altered in *Zscan2−/−* mice maintained under homeostatic conditions, at 12 weeks following low dose busulfan treatment, regeneration of spermatogenesis was significantly impaired compared with *Zscan2+/−* control counterparts (Figures 5B–E, Supplementary Figure S7; “untreated” control is from Zscan2+/− mice). We found that the testis-to-body weight ratio was significantly (*P* < 0.05) reduced by 36 ± 4.7% (*n* = 4 different mice) for *Zscan2−/−* mice compared with *Zscan2+/−* mice (Figure 5B). In accordance, relative sperm concentration in the cauda epididymis was also significantly (*P* < 0.05) reduced by 66 ± 13.5% (*n* = 4 different mice) in *Zscan2−/−* males (Figure 5C). Moreover, the percentage of seminiferous tubules lacking spermatogenesis in cross-sections of testes from *Zscan2−/−* mice was greater by 3-fold compared with +/− counterparts (Figure 5D and E). Finally, in assessing the number of undifferentiated spermatogonia (PLZF+) per tubule, there was a 70% reduction in *Zscan2−/−* mice (*P* < 0.001, *n* = 4 different mice, Figure 5F and G), suggesting that regeneration of the undifferentiated population is indeed defective in the knockout line.

**Figure 6:**
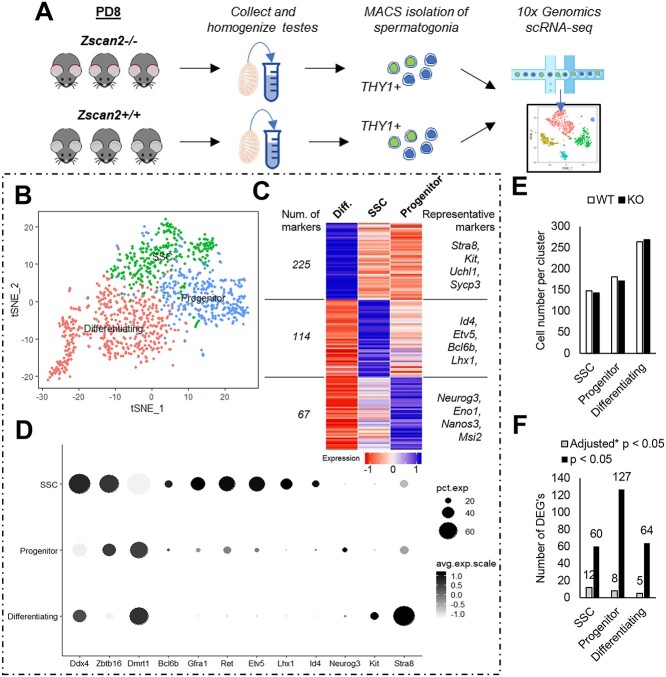
scRNA-seq analysis of ZSCAN2 deficient pup spermatogonia. (*A*) Schematic of the experimental scheme. (*B*) Merged tSNE analysis of germ cells from *Zscan2+/+* and *Zscan2−/−* mice to delineate spermatogonial stem cells (SSCs), progenitor spermatogonia, and differentiating spermatogonia subpopulations based on the expression of distinguishing markers. (*C*) Heatmap representation of differential marker gene expression for SSC (*Id4, Etv5, Bcl6b, Lhx1*), progenitor (*Neurog3, Eno1, Nanos3, Msl2*), and differentiating (*Stra8, Kit, Uchl1, Sycp3*) spermatogonial subsets. (*D*) Dot plot depiction of differentially expressed genes across spermatogonial subsets. *Ddx4*, *Zbtb16*, and *Dmrt1* are pan-germ cell markers; *Bcl6b*, *Gfra1*, *Ret*, *Etv5*, *Lhx1*, and *Id4* are SSC markers; *Neurog3* is a progenitor marker, and *Kit* and *Stra8* are expressed in differentiating spermatogonia populations. Dot color represents level of expression and dot size represents percentage of cells in a cluster in which expression could be detected. (*E*) Distribution of cells across clusters from *Zscan2+/+* and *Zscan2−/−* mice. (*F*) Number of differentially expressed genes (DEGs) in SSC, progenitor and differentiating spermatogonial subpopulations from *Zscan2−/−* mice compared with *Zscan2+/+* mice. Data are DEGs with *P* < 0.05 and adjusted *P* < 0.05 (based on Bonferroni correction using all genes in the dataset).

Lastly, we aimed to begin defining a mechanism of action for ZSCAN2 in regulating regenerative capacity of the spermatogenic lineage. To address this, scRNA-seq analysis was conducted on the THY1+ undifferentiated spermatogonial populations isolated from PD8 testes of *Zscan2+/+* (*n* = 3) and *Zscan2−/−* (*n* = 3) mice (depicted in schematic in Figure 6A). Analysis of scRNA-seq was conducted on a merged dataset containing 593 *Zscan2+/+* and 583 *Zscan2−/−* spermatogonia. Each cell had an average of 10,818 unique molecular indices (UMIs) and 2682 genes detected. Unsupervised clustering projected onto *t*-distributed stochastic neighbor embedding (tSNE) analysis plots revealed 7 populations (Supplementary Figure S8A) that could be categorized into SSC, progenitor, and differentiating spermatogonial pools based on expression of known markers (Figure 6B–D, Supplementary Figure S8B and C and listed in Supplementary Dataset S1). Effective clustering of these three populations can be appreciated by distinct gene expression profiles represented via heatmap in Figure 6C. In observing distribution of *Zscan2+/+* and *Zscan2−/−* cells, there was no bias in terms of contribution to specific clusters (Figure 6E). Comparisons of these datasets did however identify a number of DEGs between *Zscan2+/+* and *Zscan2−/−* populations in stem cell, progenitor and differentiating clusters (Figure 6F, Supplementary Dataset S1); 79.3% of DEGs exhibited downregulated expression in *Zscan2−/−* populations, while only 20.7% were upregulated. Gene ontology (GO) analysis was used to determine the top 5 biological processes represented in downregulated and upregulated DEG lists (Supplementary Figure S8D, only two biological processes were found to be enriched in the “upregulated” DEG list). Similarities existed across the three spermatogonial subpopulations in that “translation” was consistently downregulated, and as such, an interacting network of proteins involved in translation was found to be common to the list of “top 10 DEGs” in each category (Supplementary Figure S8E). GO analysis of “Functional categories” also revealed that proteins involved in acetylation were commonly downregulated across stem cell, progenitor and differentiating subsets (Supplementary Figure S8D). GO terms that were unique to DEGs in the SSC population included protein import into the nucleus, and ubiquitin conjugation (both downregulated), as well as cell redox homeostasis (upregulated) (Supplementary Figure S8D). To provide further representation of the summarized data for DEGs, additional graphical visualization was conducted for Ubiquitin-52 Amino Acid Fusion Protein (*Uba52*); a protein involved in regulation of translation, chromatin modification, and ubiquitin conjugation. In the *Zscan2+/+* dataset, dot plots depict highest levels of *Uba52* expression in the SSC population, with step-wise reductions in expression upon the progenitor and differentiating transitions; a trend that mimics expression of *Zscan2* itself (Supplementary Figure S8F). Subsequently, in *Zscan2−/−* cells, the decline in *Uba52* expression is appreciable across each spermatogonial subpopulation (Supplementary Figure S8F). In considering the possibility that diminished *Uba52* expression and impairment of associated pathways may be linked with reduced capacity for regeneration of the germline in *Zscan2−/−* mice, it is important to note that, like *Zscan2*, siRNA targeted knockdown of *Uba52* in primary cultures of undifferentiated spermatogonia caused a significant (*P* < 0.05) reduction in the percentage of ID4-eGFP^Bright^ cells (Supplementary Figure S8G, pup culture).

## Discussion

Proper control of regenerative capacity in spermatogonia is critical for the continuation of spermatogenesis in homeostatic conditions and following gonadotoxic insult. In the current study, we developed a novel high-throughput screening approach using primary cultures of spermatogonia derived from *Id4-eGfp* transgenic mice to generate an extensive new repository of information for individual transcription factors. This information has major utility for modeling potential networks and pathways that regulate activities of the undifferentiated spermatogonial population which possesses the regenerative capacity for the spermatogenic lineage. In particular, using this database we uncovered previously unappreciated roles for the CREB acetylation complex and ZSCAN2 as important influencers of spermatogenic stem cell function, thus validating its utility.

The screening methodology devised for the current study provides a novel means to rapidly and in a high throughput manner assess fluctuations in the stem cell capacity of the undifferentiated spermatogonial population. As such, the approach has utility as an alternative to the only other verified method for quantitatively assessing alterations in stem cell function of an experimental population, spermatogonial transplantation [6, 7, 39]. The utilization of primary cultures of spermatogonia in our pipeline makes it particularly useful for large scale assessment of molecular pathways and gene families by facilitating the expansion of cell populations that are lowly abundant in vivo, while concomitantly retaining the distinct functional properties and gene expression signatures of these cells. Beyond this, adaptations on the methodology may be valuable for prescreening of toxic effects on the undifferentiated spermatogonial pool in a drug development pipeline, or for large-scale exploration into the role of genes that regulate other key processes influencing undifferentiated spermatogonial functions such as metabolism and epigenetic programming.

The extensive repository of information generated using our high-throughput pipeline provides a key resource for the field. Indeed, the collective outcome from siRNA knockdown of over 1400 transcription factors in this study revealed previously unappreciated individual factors and networks that are predicted to be fundamental for regenerative capacity in spermatogonia. Importantly, the validity of results produced using this approach have been demonstrated within the parameters of our siRNA screen via the successful identification and classification of transcription factors that have been previously characterized to be required for maintenance of stem cell function, such as RB1 [20], FOXO1 [40], CHD4 [19], and GLIS3 [41], and conversely, the identification of confirmed negative regulators of stem cell capacity such as FBXW7 [42]. Indeed, 45 of 547 genes identified using this screen have been previously found to be differentially expressed between SSC and progenitor populations via RNA-seq analysis (using the stringent cutoff of *P* < 0.001) [10]. However, our results also reveal the complexities of regulatory loops controlling gene expression in SSCs, which can complicate interpretation of the screen output. Specifically, siRNA targeting of endogenous *Id4* and *Etv5* resulted in upregulation of *Id4-eGfp* transgene expression, despite their known requirement for SSC maintenance [9, 32]. Given that endogenous ID4 exerts autoregulatory repression [43] via a regulatory region that is also shared by the transgene (E-box region) [9], loss of endogenous ID4 likely releases a “handbrake” on transgene expression, causing it to increase. Because ETV5 has been demonstrated to influence dimerization of basic helix-loop-helix (bHLH) factors [44], it is possible that *Etv5* knockdown thus impairs the capacity for ID4 to sequester bHLH binding partners that target its own E box promoter, releasing them to initiate upregulated transcription of the *Id4-eGfp* transgene. Regardless of these nuances in the siRNA screen readout, we predict that the database produced will be useful for investigators as a starting point for assessing whether a transcription factor of interest may influence either maintenance of stem cell function or the stem cell-to-progenitor transition in spermatogonia. Also, this database provides a means to identify important regulatory genes that may not be differentially expressed between spermatogonial populations at the transcript level, but instead may be posttranscriptionally regulated or posttranslationally modified to elicit alternate functions in SSC and progenitor populations.

An intriguing outcome of the siRNA screen was flagging components of the CREB acetylation complex as potential regulators of spermatogenic stem cell function. In other cell types, this complex influences the acetylation of histones; facilitating transcription factor binding and thus transcriptional activation. Primarily, the acetyltransferase enzyme CREBBP was identified in our “top 5” list of factors whose knockdown resulted in reduced MFI. We confirmed that loss of CREBBP and EP300 acetyltransferase expression, as well as knockdown of the binding partner SRCAP that upregulates activity of the complex [45], instigated a transition from self-renewal to a transit-amplifying progenitor state. In addition, knockdown of *Srcap* in primary cultures of spermatogonia led to a reduction in H4K16ac levels specifically in the ID4-eGFP^Bright^ population along with a decrease in expression of both *Id4* and *Zscan2*. These findings suggest that the CREB complex mediates high levels of H4K16ac in spermatogonia to promote expression of key genes that drive stem cell maintenance, potentially in a manner similar to the role in regulating self-renewal of embryonic stem cells [46]. Also, these results integrated well with the identification of FOXO, Notch, and HIF-1 signaling as potential regulators of regenerative capacity in spermatogonia. Although the role of FOXO signaling in regulation of spermatogenic stem cell function is well established, with conditional knockout mouse models demonstrating that FOXO1 is important for male germline maintenance [40], the potential importance of the CREB protein complex in facilitating the regulatory action of FOXO1 has not previously been appreciated. Interaction of FOXO1 with CREBBP and EP300 has been proposed to be indispensable for FOXO1-driven transcription (reviewed in [47]). In support of this concept, we found that individual knockdown of FOXO1, CREBBP, and EP300 elicited a similar response of reduced stem cell content in primary cultures of undifferentiated spermatogonia.

Another key outcome of our use of the screen to identify new factors regulating spermatogonial functions was the flagging of *Zscan2*. Building upon findings from a descriptive study published three decades ago [35], our gene expression analyses revealed that *Zscan2* is a testis-enriched transcription factor in the mouse with elevated expression in the undifferentiated spermatogonial pool. Beyond the seemingly testis specificity in the mouse, we found that *Zscan2* gene structure and expression is evolutionarily conserved, inferring a potentially important role in sustaining male fertility for several mammalian species. Importantly, we report the first generation of a *Zscan2* null mouse model to study its role in spermatogenesis. Despite previous reports of *Zscan2* expression in the developing embryo (from E14.5 onwards in the mouse) [35], the null mouse model exhibited no aberrations in germ cell specification or testis development, suggesting an indispensable role in these formative processes. In assessing spermatogenesis in adulthood, results of a breeding trial suggested that ZSCAN2 is dispensable for steady-state spermatogenesis. However, the context in which the animals were maintained in a highly controlled environment without exposures to physiological stressors or cytotoxic insults likely does not reflect the context in which animals and humans typically live, thus a true role in maintaining integrity of the spermatogenic lineage could not be fully appreciated. Indeed, we discovered that normal regeneration of spermatogenesis following exposure to a cytotoxic insult (i.e., the chemotherapeutic agent busulfan) was compromised in a ZSCAN2 deficient state. As low-dose chemotherapeutic exposure has been demonstrated previously to mimic the effects on spermatogenesis elicited by other stressors such as famine [48], it is plausible that ZSCAN2 provides an evolutionary advantage in terms of fertility preservation in times of environmental strain.

Collectively, outcomes of the current study describe a novel high-throughput methodology for screening of candidate factors that influence regenerative capacity of the undifferentiated spermatogonial population and provide a database of information for the male germline stem cell field to fuel future investigations on transcription factor networks that regulate spermatogonial biology. Our investigation has identified a number of previously unappreciated factors and networks that can be predicted to regulate regenerative fitness. In particular, we have uncovered a potential role for the CREB complex in promoting H4K16ac levels to upregulate expression of genes that influence the stem cell state of spermatogonia, including *Zscan2* which encodes for a novel, highly conserved, testis-enriched transcription factor whose actions underlie efficient regeneration of the spermatogenic lineage in vivo. We have demonstrated that restoration of spermatogenesis is severely impaired in male mice with ZSCAN2 deficiency following exposure to the clastogen busulfan, a finding that may have implications in a clinical medicine setting for advising cancer patients on risk of fertility defects following treatment and help explain idiopathic infertility of men, domestic animals, wildlife, or endangered species that may be caused by environmental stressors.

## Supplementary Material

Supp_figures_ioac048

Supplemental_tables_ioac048

Supplemental_dataset_S1_ioac048

## Data Availability

Transcriptome (scRNA-seq) data generated and analyzed in this study are available from the GEO database (accession number GSE197497).
