# Supplementary figures and images for: A novel high throughput screen to identify candidate molecular networks that regulate spermatogenic stem cell functions
Source: Biol Reprod. 2022 Mar 4;106(6):1175–90. doi: 10.1093/biolre/ioac048 (PMC9198950; doi:10.1093/biolre/ioac048)

# Figure S1

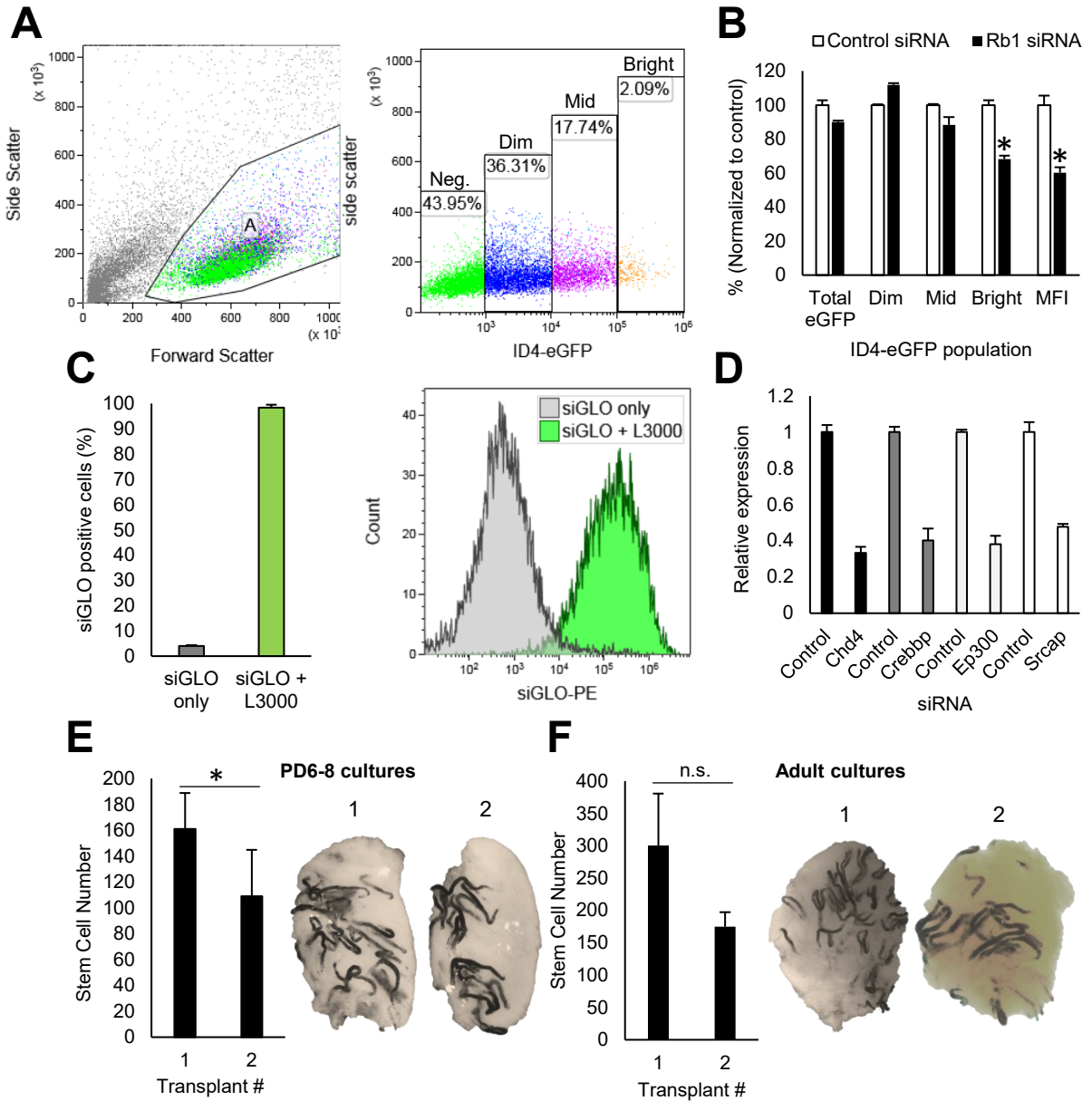

**Figure S2**

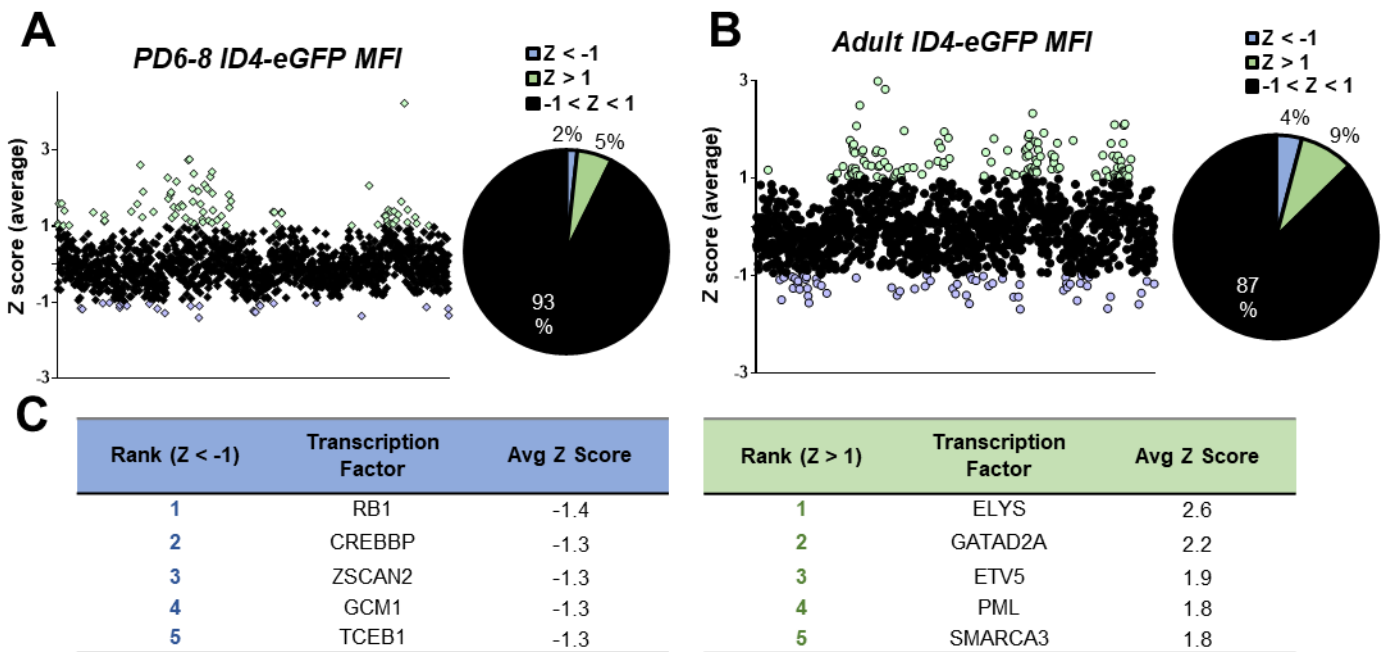

# Figure S3

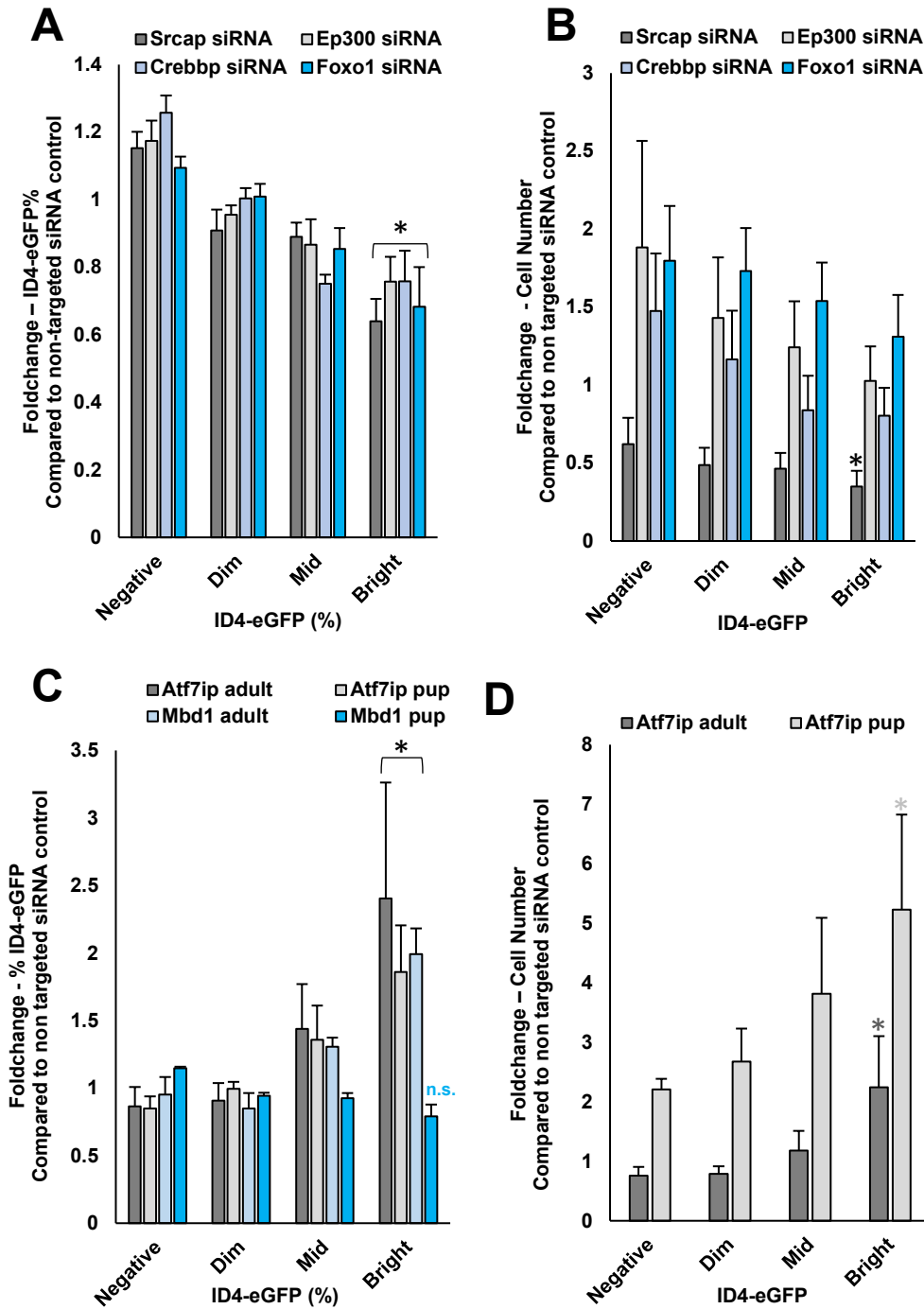

Figure S4

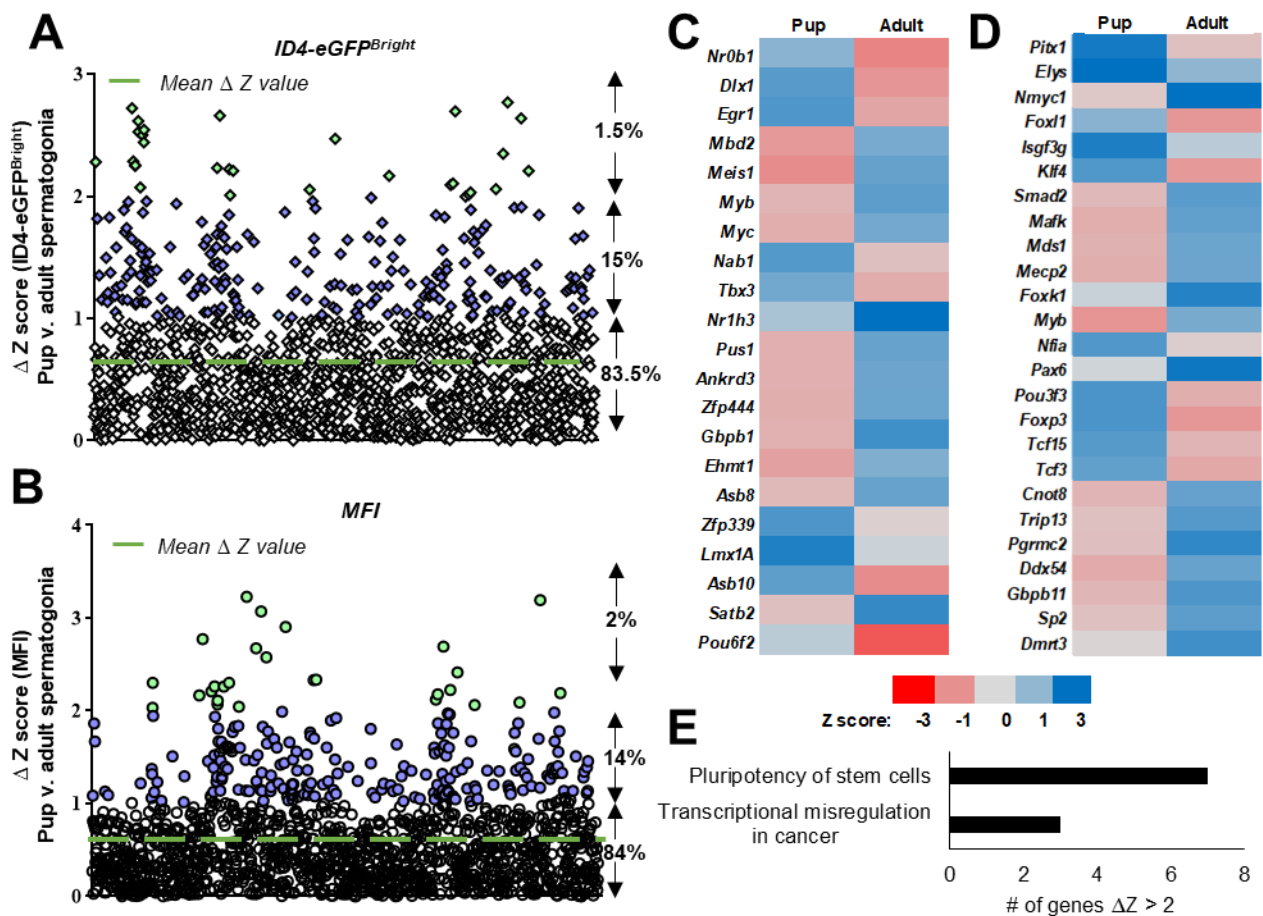

Figure S5

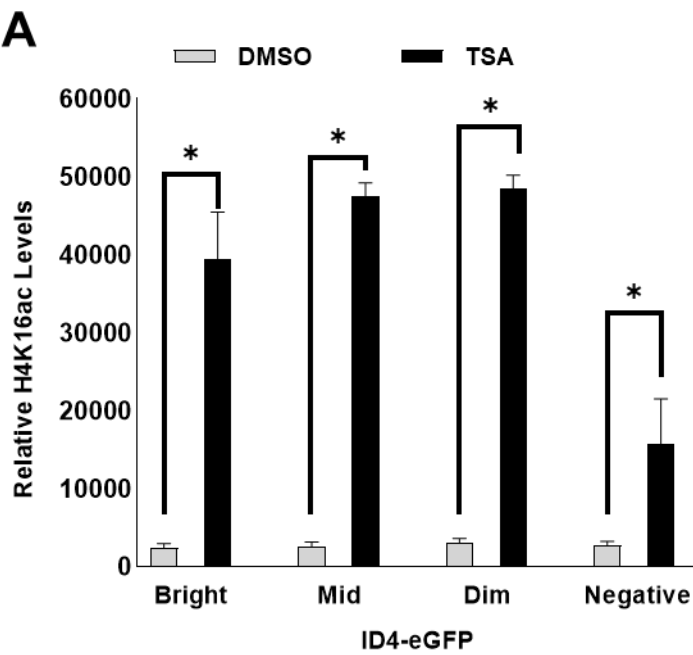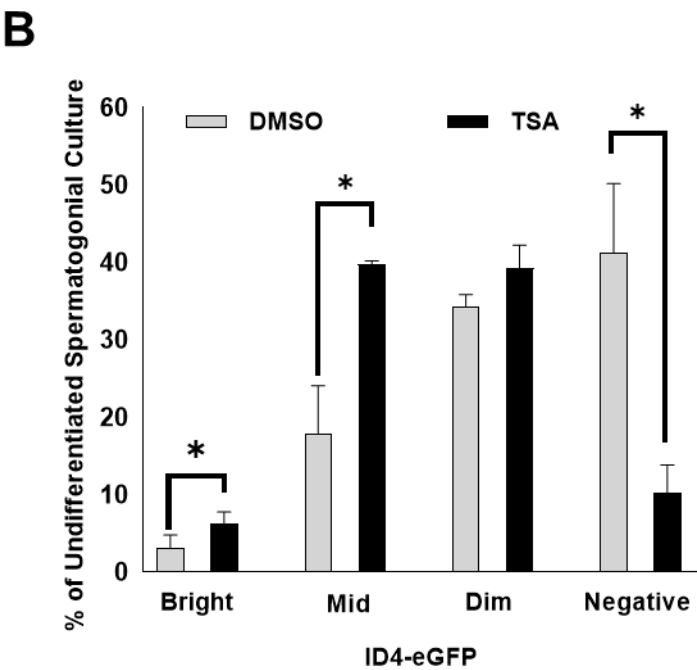

Figure S6

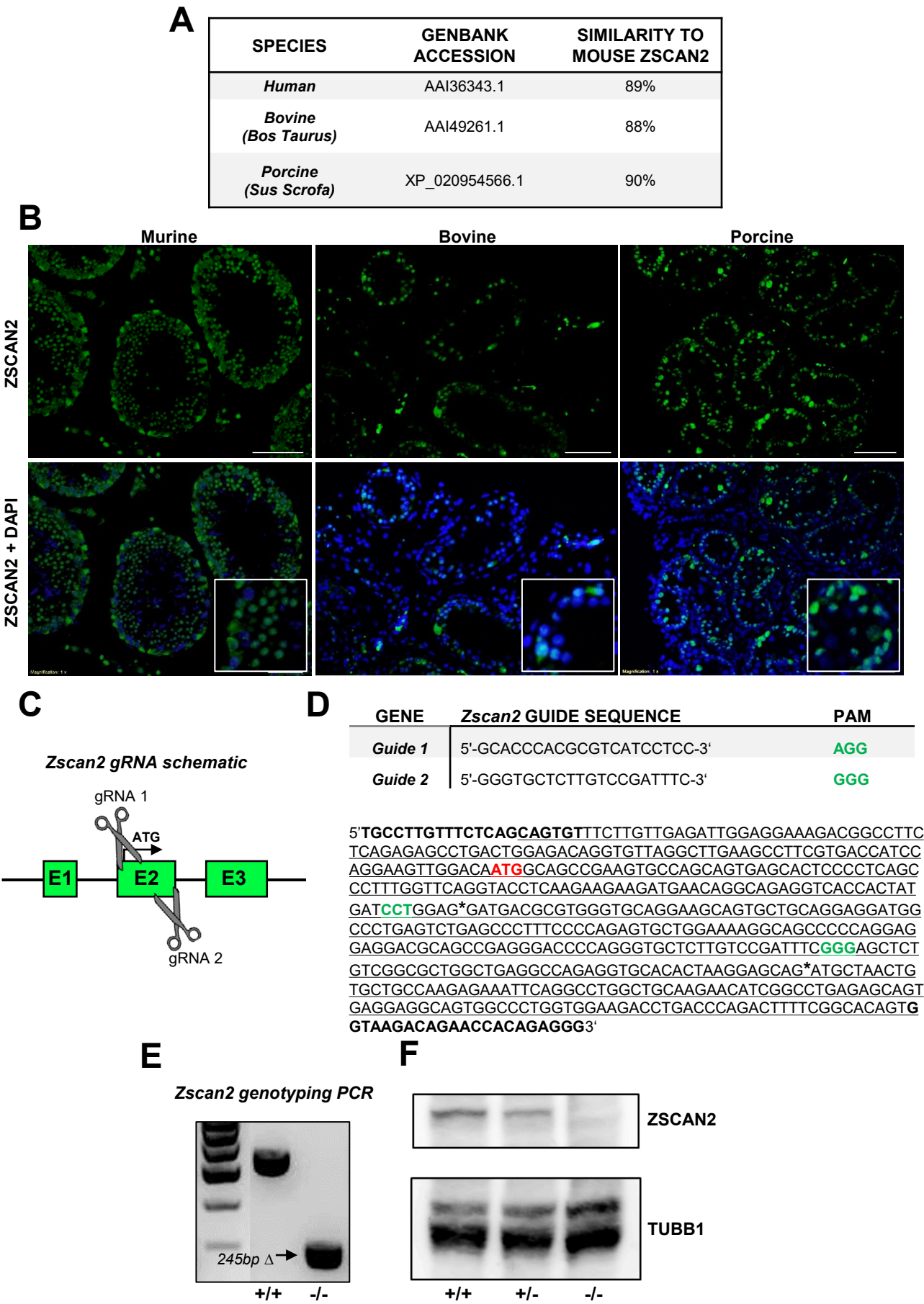

Figure S7

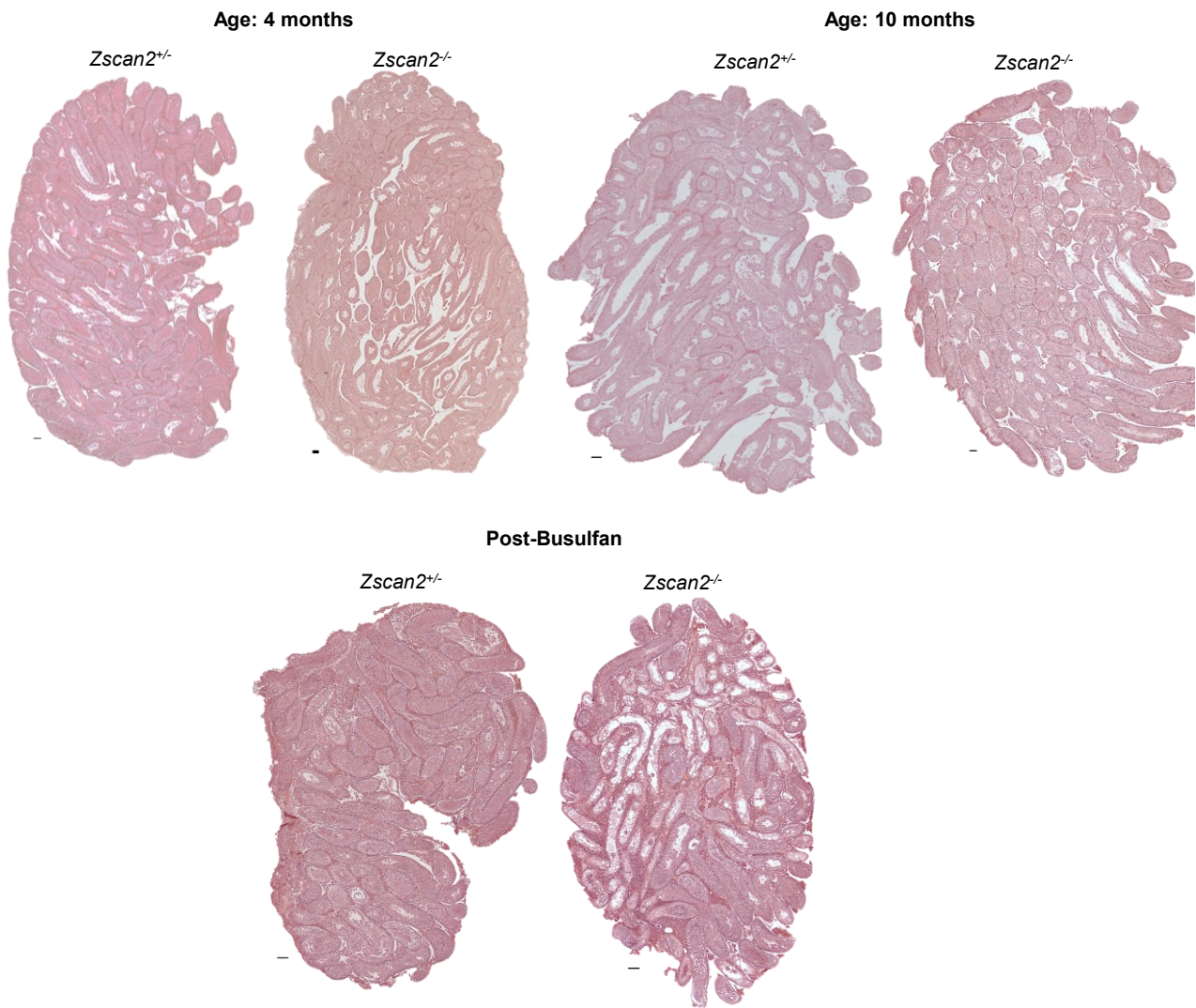

# Figure S8

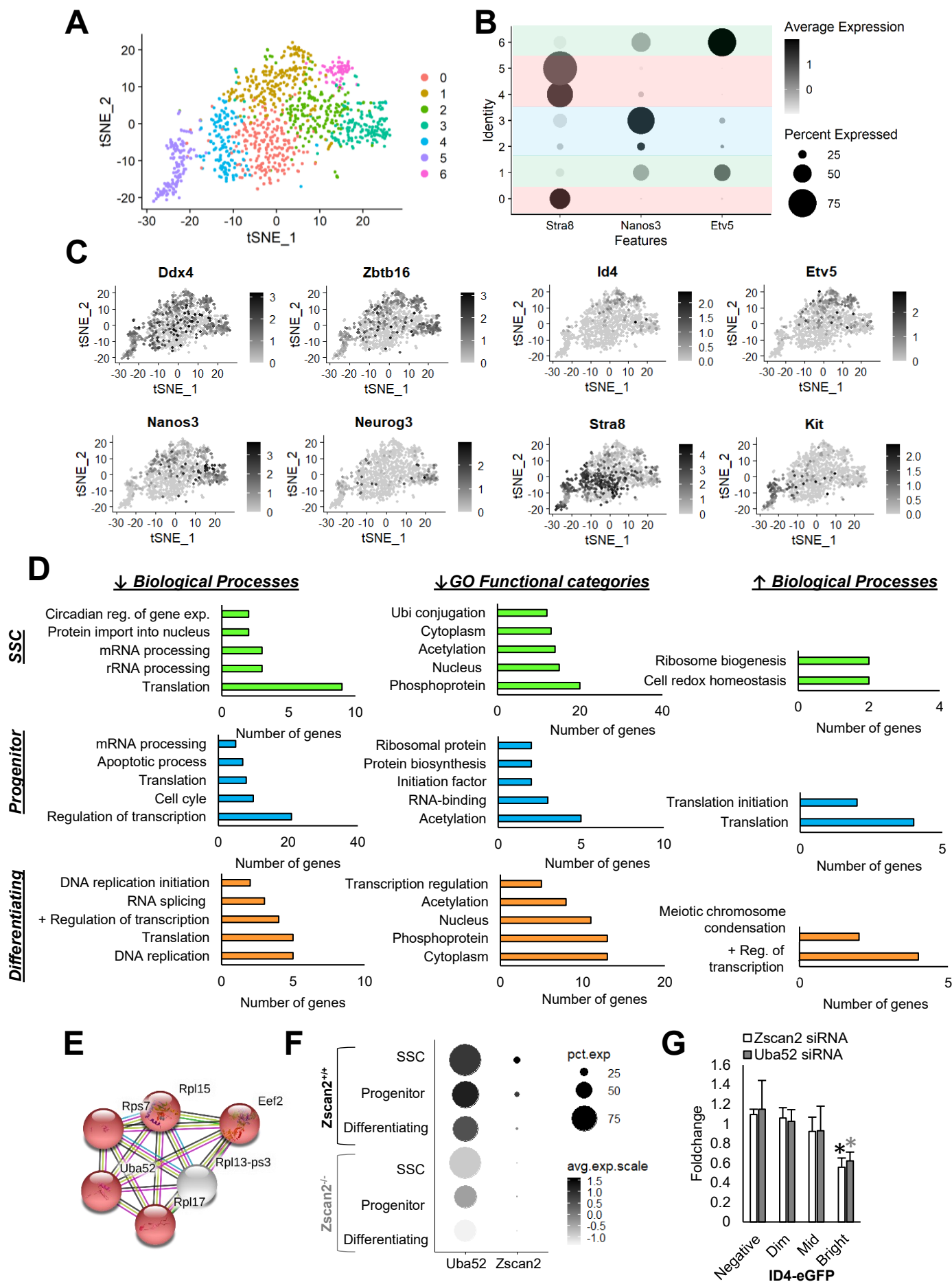

Supplement: Supp_figures_ioac048 [file supp_figures_ioac048.pdf]
