# Supplementary material for: A novel high throughput screen to identify candidate molecular networks that regulate spermatogenic stem cell functions
Source: Biol Reprod. 2022 Mar 4;106(6):1175–90. doi: 10.1093/biolre/ioac048 (PMC9198950; doi:10.1093/biolre/ioac048)
Supplement: Supplemental_tables_ioac048 [file supplemental_tables_ioac048.pdf]

Table S1: Primers used for genotyping and qRT-PCR analysis

GENE	PRIMER	PRODUCT SIZE
Zscan2	**(Genotyping)**	
	F-5'- TGCCTTGTTTCTCAGCAGTCT -3‘	
	R-5'- CCCTCTGTGGTTCTGTCTTACC -3‘	543 bp
	**(qRT-PCR)**	
	Taqman probe Mm_00437360_m1	70 bp
Id4	**(qRT-PCR)**	
	Taqman probe Mm_00499701_m1	102 bp
Crebbp	**(qRT-PCR)**	
	Taqman probe Mm01342452_m1	66 bp
Ep300	**(qRT-PCR)**	
	Taqman probe Mm00625535_m1	145 bp
Srcap	**(qRT-PCR)**	
	Taqman probe Mm00613213_m1	75 bp
Rps2	**(qRT-PCR)**	
	Taqman probe Mm01971861_g1	62 bp

Table S2: Antibodies used for Western blotting and Immunofluorescence

TARGET	ANTIBODY	CONCENTRATION	ISOTYPE
ZSCAN2	Rabbit pAb to ZSCAN2<br>Sigma/Atlas antibodies #HPA024331	WB: 1/4000<br>IF: 1/500	IgG
H4K16ac	Rat mAb to H4 Lys 16 acetylation<br>Cell Signaling Tech #13534	FCA: 1/500	IgG
B-TUBULIN	Rabbit pAb to beta-Tubulin<br>Novus Biologicals #NB600-936	WB: 1/4000	IgG
PLZF	Mouse mAB to PLZF (ZBTB16)<br>Santa Cruz Biotechnology #sc-28319	IF: 1:200	IgG
SOX9	Goat pAB to SOX9<br>R&D Systems #AF3075	IF: 1:50	IgG
